# Novel strategies in scalp expansion: improvements and applications of tissue expanders

**DOI:** 10.1093/burnst/tkae002

**Published:** 2024-04-08

**Authors:** Fangzhou Xie, Jiaqi Qin, Bin Fang, Shuangbai Zhou, Ru-Lin Huang, Qingfeng Li, Rao Fu, Yun Xie

**Affiliations:** Department of Plastic and Reconstructive Surgery, Shanghai Ninth People’s Hospital, Shanghai Jiaotong University School of Medicine, 639 Zhizaoju Road, Shanghai 200011, China; Department of Plastic and Reconstructive Surgery, Shanghai Ninth People’s Hospital, Shanghai Jiaotong University School of Medicine, 639 Zhizaoju Road, Shanghai 200011, China; Department of Plastic and Reconstructive Surgery, Shanghai Ninth People’s Hospital, Shanghai Jiaotong University School of Medicine, 639 Zhizaoju Road, Shanghai 200011, China; Department of Plastic and Reconstructive Surgery, Shanghai Ninth People’s Hospital, Shanghai Jiaotong University School of Medicine, 639 Zhizaoju Road, Shanghai 200011, China; Department of Plastic and Reconstructive Surgery, Shanghai Ninth People’s Hospital, Shanghai Jiaotong University School of Medicine, 639 Zhizaoju Road, Shanghai 200011, China; Department of Plastic and Reconstructive Surgery, Shanghai Ninth People’s Hospital, Shanghai Jiaotong University School of Medicine, 639 Zhizaoju Road, Shanghai 200011, China; Department of Plastic and Reconstructive Surgery, Shanghai Ninth People’s Hospital, Shanghai Jiaotong University School of Medicine, 639 Zhizaoju Road, Shanghai 200011, China; Department of Plastic and Reconstructive Surgery, Shanghai Ninth People’s Hospital, Shanghai Jiaotong University School of Medicine, 639 Zhizaoju Road, Shanghai 200011, China

## To the Editor

Tissue expanders have been widely used in plastic and reconstructive surgery. They induce skin regeneration through mechanical tension and generate new tissue to repair and reconstruct soft tissue defects throughout the body [1]. The scalp, with its rich blood supply, vigorous cell proliferation and abundant hair follicle stem cells, can yield a significant amount of hair-bearing skin after expansion [2]. It has become an essential donor site for head and face reconstruction. Similar to standard expanders, those for scalp expansion comprise an expansion sac and an injection pot but face three main challenges. (1) Expander undersurface rupture. During scalp expansion, continuous mechanical tension may cause bone remodeling of the skull bone, with bone resorption and matrix deposition at the compression site. Together with periosteal reactive hyperplasia around the expander, this may lead to ‘bone spur’-like structures on the bone surface [3]. These spurs, along with the expansion sac’s movement, can cause expander rupture due to friction, leading to expansion failure. (2) Early closure of the expansion sac. In the early postoperative period, due to the poor elasticity of the scalp compared with other parts of the skin, a small amount of water injection may cause the patient to feel noticeable swelling or pain [4]. However, insufficient or delayed filling can lead to spontaneous sac closure or wall thickening. Injecting a small volume of water during or immediately after surgery compresses wound blood vessels, reducing bleeding and enlarging the sac. However, distinguishing this from a postoperative haematoma is challenging. Therefore, ensuring no haematoma post-extubation and timely sac filling is crucial to maintaining its post-surgical size. (3) Folding, flipping and displacement of the expander. During the placement surgery of the expander, a subcutaneous pocket consistent with the shape of the expander is generally separated, which is 1 cm larger than the sac circumference. The expander, with its base facing downward, is positioned to ensure stability and proper skin expansion. However, the scalp’s high tenacity and tension mean early water injections might not fully fill the sac. Because of this, the expander often experiences sac wall folding (this may cause thinning and necrosis of the skin), flipping (this may cause the base of the expander to crease the skin) and displacement, meaning the expansion area may not meet the original surgical design and other problems may also arise [5].

The above three problems often prevent the scalp from reaching the desired volume and shape, and may even cause rupture and failure. To enhance expansion efficacy and mitigate associated risks, we introduced three novel tissue expander designs and conducted clinical case validation.

### Design innovations and clinical validation

**Figure 1:**
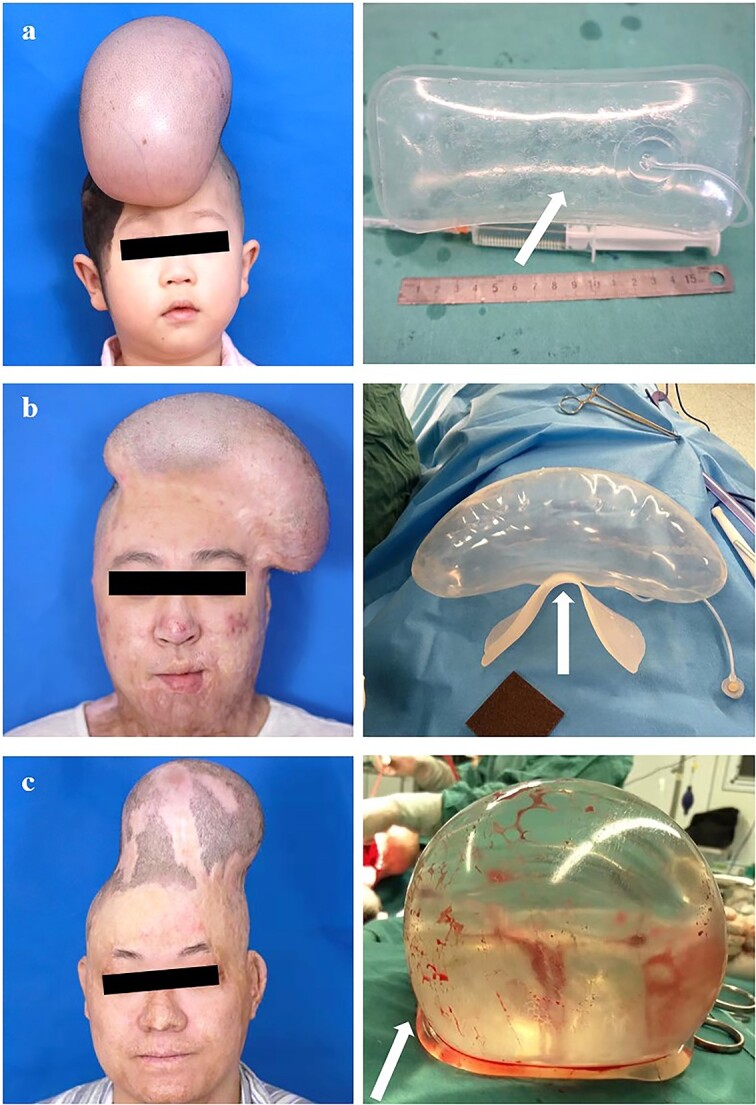
Three novel tissue expanders are used for scalp expansion: left, clinical cases; right, the tissue expanders. (**a**) Base-thickened tissue expander (arrow points to the thickened base). (**b**) Tissue expander with a base plate (arrow points to the base plate). (**c**) Tissue expander with a bottom cushion sac (arrow points to the cushion sac)

These improvements are mainly put into three categories, as follows: thickening the base, adding a base plate and incorporating a cushioning sac at the base. The base-thickened tissue expander consists of an expansion sac with a silicone-thickened base, a water injection pot and a connecting tube; the sac’s base is 2–3 times thicker than its wall (Figure 1a). The tissue expander with a base plate includes similar components, plus connecting rings and a curved, rough-bottomed base plate connected to the sac (Figure 1b). The tissue expander with a bottom cushion sac features an expansion sac, a primary and a secondary injection pot, respective tubes, and an airbag cushion sac with a rough underside attached to the sac’s base for inflation (Figure 1c). Both the base plate and cushion sac designs aim to prevent direct contact between bone spurs and the expander sac, reducing the risk of flipping and shifting.

We conducted clinical validation of each of the three tissue expanders in our department. All patients with head and face lesions affecting aesthetics or function underwent reconstruction, completing thorough medical evaluations. The choice of tissue expander is based on the patient’s age, surgical design and the patient’s willingness. After implantation, patients were monitored for the condition of the expander and expanded scalp. Each of the three tissue expanders was implanted in three patients. During the scalp expansion process, no problems like base rupture, flipping and displacement, or early closure were observed (Figure 1). After the scalp expansion, surgeries were conducted to remove the expander, excise the lesion and perform a local skin flap transfer. All patients are currently recovering well.

### Clinical insights and discussion

After clinical implementation, we further discussed and summarized our experiences. The base-thickened tissue expander’s key enhancement is its silicone-thickened base, which diminishes the risk of bone spurs penetrating the dilatation capsule. However, as it expands, this thickened base hinders the expander’s base efficiency, leading to more outward bulging and increased height with an almost unchanged base area. Based on clinical analysis, we further developed two new designs: the tissue expander with a base plate and the tissue expander with a bottom cushion sac.

The former includes a silicone base plate fixed with connecting rings beneath the sac, creating a barrier against bone spurs and preventing sac rupture. The concave arc-shaped base plate enhances contact with subcutaneous tissues, preventing sac flipping or shifting. Once bonded with the subcutaneous pocket, it creates a buffer space, stabilizing the sac even during active expansion. The base plate increases the overall thickness and rigidity of the expander’s base. It helps to compress the wound blood vessels and enlarge the capsular cavity, excluding early complications such as haematoma and effectively avoiding spontaneous closure or contracture of the dilated capsular cavity. Additionally, it stops the expander from sinking into soft tissue, promoting upward expansion and skin regeneration. The connecting rings maintain the expander’s shape and position during early water injection.

The latter design incorporates a cushion sac, preventing direct contact between the bone spur and the expansion sac, thus reducing rupture risk. Even if the cushion ruptures, its triple-layered wall acts as an additional barrier against punctures. The cushion’s rough undersurface further minimizes puncture risk. During surgery, the uninflated airbag ensures easy placement. Post-surgery, inflating the airbag prevents shifting and flipping of the expander, and helps avoid early sac closure and wall contracture. The distinct texture of the cushion sac aids in differentiating it from haematoma, facilitating complication assessment. Additionally, the cushion sac prevents the expander from sinking into soft tissue, promoting upward expansion and efficiency. If the sac’s base and wall separate, saline flows into the cushion sac, further reducing rupture risk.

The three types of tissue expanders mentioned above can address the rupture problems caused by friction between bone spurs and the sac to a certain degree. Considering implantation ease, manufacturing cost and care ease during the expansion phase, the tissue expander with a base plate is the most optimal (Table 1). More importantly, clinicians must select the most suitable expander tailored to each specific clinical scenario to ensure safe and effective scalp expansion. This research contributes to the field of scalp expansion and serves as a reference for future applications.

**Table 1:** Comparison of three types of scalp tissue expanders

**Type**	**Advantage**	**Drawback**
Base-thickened tissue expander	Avoids sac undersurface rupture when the volume of injection is small.	Higher risk with larger injection volume. Low expansion efficiency. Unable to resolve inversion, shift and early closure.
Tissue expander with a base plate	Effectively solves the above problems. High expansion efficiency.	The overall thickness increases the difficulty of implantation.
Tissue expander with a bottom cushion sac	Cushion sac design, lowest risk of rupture. Effectively solves the above problems. High expansion efficiency.	The oversized cushion sac significantly increases the difficulty of implantation. High production cost.

## Funding

Funding was provided by Clinical Research Program of Ninth People’s Hospital, Shanghai JiaoTong University School of Medicine (JYLJ202103); The Two-hundred Talent (20191916); and the Shanghai Clinical Research Center of Plastic and Reconstructive Surgery supported by the Science and Technology Commission of Shanghai Municipality (22MC1940300).

## Authors’ contributions

FZX and JQQ extracted data from the included studies and wrote the manuscript. BF conducted the literature review and background research. SBZ and RF conducted follow-up and information collection for patients. YX and R-LH designed the expanders. YX and QFL designed the study and provided guidance for preparation of the manuscript.

## Ethics approval and consent to participate

The study was conducted in accordance with the guidelines of the Helsinki Declaration as revised in 2013. The study protocol was agreed by the local ethics committee of the Shanghai Ninth People’s Hospital. All patients in our department provide written consent for the utilization of the data obtained from their surgeries, follow-up visits and for non-identifiable photographs.

## Conflict of interest

None declared.
